# Connecting primary care patients to community-based physical activity: a qualitative study of health professional and patient views

**DOI:** 10.3399/bjgpopen20X101100

**Published:** 2020-07-22

**Authors:** Sharon Ann Carstairs, Rayna H Rogowsky, Kathryn B Cunningham, Frank Sullivan, Gozde Ozakinci

**Affiliations:** 1 Population and Behavioural Sciences, School of Medicine, University of St Andrews, St Andrews, UK

**Keywords:** Physical activity, health promotion, social prescribing, primary health care, consultation, motivation

## Abstract

**Background:**

Inconclusive evidence supporting referrals from health professionals to gym-based exercise programmes has raised concern for the roll-out of such schemes, and highlights the importance of developing links between healthcare settings and community-based opportunities to improve physical activity (PA) levels.

**Aim:**

This study aimed to identify methods, and explore barriers and facilitators, of connecting primary care patients with PA opportunities from the perspectives of both health professionals (HPs) and patients, using the example of jog**s** **cotland**.

**Design & setting:**

An exploratory study utilising semi-structured interviews with primary care patients (*n* = 14) and HPs (*n* = 14) from one UK NHS board was conducted.

**Method:**

Patient and HP transcripts were analysed separately using thematic analysis. Potential methods of connection were identified. The Capability, Opportunity, Motivation, behavioural (COM-B) model and theoretical domains framework (TDF) were employed to facilitate identification of barriers and facilitators for connecting primary care to community jog**scotland** groups.

**Results:**

Three methods of connecting patients to community-based groups were identified: informal passive signposting, informal active signposting, and formal referral or prescribing. Barriers and facilitators for patient connection fell into five TDF domains for HPs and two COM-B model components for patients.

**Conclusion:**

For patients, HPs raising the topic of PA can help to justify, facilitate, and motivate action to change. The workload associated with connecting patients with community-based opportunities is central to implementation by HPs. Integrative resource solutions and social support for patients can provide a greater variety of PA options and the vital information and support for connecting with local opportunities, such as jog**scotland**.

## How this fits in

Previous quantitative and qualitative studies in the UK have highlighted many barriers to PA promotion in healthcare settings. This study adds both HP and patient views on the different methods of promotion that could be employed for connecting primary care patients to community-based opportunities, such as jog**scotland**. It utilises psychological frameworks of behaviour to provide a unique understanding of the views of both parties on PA connection, and the solutions that could be implemented within primary care to overcome barriers.

## Introduction

The role of PA promotion as a ‘best buy’ in health behaviour interventions has long been advocated by global public health strategic plans.^1,2^ In 2006, public health guidance^3^ endorsed four common methods of promoting increased PA levels of the population in the UK: brief interventions in primary care, exercise referral schemes, use of pedometers, and community-based programmes. These preventative strategies have continued in the development of more recent guidance^4,5^ emphasising the role of healthcare settings, such as primary care, which provide opportunistic contact with a wide range and number of patients.

Since the 1990s, HPs have been prescribing/referring patients to PA schemes,^6^ yet evidence in support of their effectiveness is weak, and often these are limited to patients with specific health conditions.^7,8^ Morgan *et al*’s systematic review^9^ concluded that the main barriers to patient adherence to referral schemes included the inconvenience of the sessions regarding cost, location, and an intimidating gym atmosphere. The format and activity at the heart of these gym-based referral schemes have also been noted in guidelines, indicating that ‘*offering alternatives to gym-based activities, that are less expensive and give a degree of personal choice, seem to improve adherence*’.^5^ Furthermore, with concern for the roll-out of referrals from primary care HPs,^10^ more recent action plans^11,12^ highlight the importance of developing links between primary care and community-based PA opportunities, broadening from the use of traditional gym-based programmes to include a range of outdoor activities, reflecting social prescribing (SP) initiatives. SP^13^ can connect patients to a wide range of existing activities in the community (for example, non-profit organisations, charities, sport clubs, and independent groups). One such group is jog**scotland**, a recreational jogging network launched in 2002^14^ (Supplementary Box S1).^15–17^

Current evidence suggests that barriers for primary care PA promotion by HPs includes: lack of time and incentive,^18^ lack of expertise, medico-legal concerns,^19^ and lack of role and responsibility.^19,20^ Furthermore, the evidence for the use and implementation of SP is inconsistent^21^ and of limited quality.^22^ There remains an evidence gap regarding the processes for delivering SP,^23^ which have been described as: signposting, direct referral, or referral to an intermediary.^24^ Investigation into the methods within these processes, and the views of both HPs and patients on these different connecting methods to community-based opportunities, is currently lacking. Using the example of jog**scotland**, this study aimed to explore both primary care HP and patient views regarding: 1) potential methods of connecting patients to community-based PA opportunities; and 2) barriers and facilitators to employing methods of connection to jog**scotland,** as a community-based opportunity.

## Method

This study reported its findings in line with the Standards for Reporting Qualitative Research checklist^25^ (Supplementary Box S2).

### Design, setting, and participants

This qualitative study was the first stage of a larger project aiming to design and test the acceptability and effectiveness of implementing a process of connecting primary care patients to local jog**scotland** groups, as a community-based approach to increase PA.

HPs with a patient-facing role within NHS general practices in the East of Scotland were invited to take part through email invitation disseminated by the NHS Research Scotland Primary Care Network to all staff in NHS Fife practices. A random sample of patient participants who were registered at a general practice in Fife were recruited using the Scottish Health Research Register.^26^ Patients were also recruited opportunistically via face-to-face advertisement at a local practice by a member of the research team. A total of 15 patient and 15 HP interviews was identified as an appropriate target sample size to provide the opportunity for the saturation of themes.^27,28^ Maximum variation sampling was used to include male and female patients, different age groups, and patients from different geographical locations across Fife. Patients who were medically advised to refrain from taking part in PA were excluded.

### Data collection

Semi-structured interviews, lasting 30–45 minutes, were conducted face-to-face at a suitable location or via telephone, between December 2018–January 2019. In line with ethical guidelines, the participant’s written informed consent was obtained prior to commencing the interview. Two interview guides (one each for HPs and patients) were developed by the research team (see researcher characteristics in Supplementary Box S2) and reviewed by jog**scotland** advisers. The guides (Supplementary Box S3) included demographic questions (age and gender) and self-reported PA levels, and were informed by the COM-B model.^29^ The COM-B model has previously assisted exploration and understanding of health-related behaviour and professional practice.^30–32^ Guides included questions about the acceptability and implementation of methods of connecting patients to jog**scotland** groups.

Interviews were conducted and digitally recorded by two female researchers (SAC, RHR), who were experienced in qualitative methods. One participant did not consent to digital recording, instead consenting for written field notes to be taken during the interview,^33^ which were later checked by the participant for accuracy.^34^ Coded audio files were securely transferred to a third-party transcription service, and were transcribed verbatim. Any identifiable information was removed from coded transcripts.

### Data analysis

Data was analysed utilising NVivo (version 11.0) software.^35^ Data analysis was conducted separately for the HPs and patient transcripts, analysing views regarding potential methods of connecting patients to the community-based PA opportunities such as jog**scotland**. To establish an understanding of the barriers and facilitators to promotion of community-based PA opportunities for HPs, the data were analysed by coding instances within the transcripts, in line with the COM-B components, and mapping onto relevant TDF domains^36^ using reflexive thematic analysis.^37^ The 14-domain TDF prompts an analysis of social, environmental, cognitive, and affective influences on HP practice.^38^ It links directly to the components of the COM-B model, and provides an integrative theoretical framework incorporating individual and organisational determinants of behaviour, useful for understanding the implementation of evidence-based practice and research.^38,39^ The data were first analysed utilising a deductive thematic analysis approach guided by the TDF domains for HPs and COM-B for patients for the emergence of themes, then analysed utilising an inductive approach to thematically generate explanatory sub-themes within the identified domains and components.

One researcher (SAC) conducted the coding of all transcripts, mapping of sub-themes, and data synthesis. A second researcher (GO), independently analysed a random sample of the interviews (20%). Differences were discussed and a consensus reached to ensure appropriateness of coding and mapping.

## Results

A total of 28 individuals (*n* = 14 HPs and *n* = 14 patients) participated in the interviews, at which point no new emerging themes were identified and thus data saturation was acknowledged. Participant demographics are presented in Table 1. HP participants included both GPs (64.3%), and practice nurses (35.7%). Self-reported PA levels identified the majority of HPs (92.9%) and patients (57.1%) as being active at least 3 days per week.

**Table 1.** Participant demographics

**Healthcare professionals (*n* = 14**)	**Patients (*n* = 14**)
**Variable**	***n*** **(** **%)**	**Variable**	***n (%*** ***)***
**Sex**		**Sex**	
Female	7 (50.0)	Female	8 (57.1)
Male	7 (50.0)	Male	6 (42.9)
**Age, years**		**Age, years**	
25–34	0 (0.0)	25–34	2 (14.3)
35–44	4 (28.6)	35–44	1 (7.1)
45–54	8 (57.1)	45–54	5 (35.7)
55–64	2 (14.3)	55–64	2 (14.3)
≥65	0 (0.0)	≥65	4 (28.6)
Role			
GP	9 (64.3)		
Practice Nurse	5 (35.7)		
**Physical** **a** **ctivity** **l** **evel (** **d** **ays per week)**		**Physical** **a** **ctivity** **l** **evel** **, d** **ays per week**	
0	0 (0.0)	0	3 (21.4)
1–2	1 (7.1)	1–2	3 (21.4)
3–4	5 (35.7)	3–4	1 (7.1)
5–6	4 (28.6)	5–6	4 (28.6)
7	4 (28.6)	7	3 (21.4)

### Connecting primary care patients to jogscotland: professional and patient views regarding potential methods

**Figure 1.**
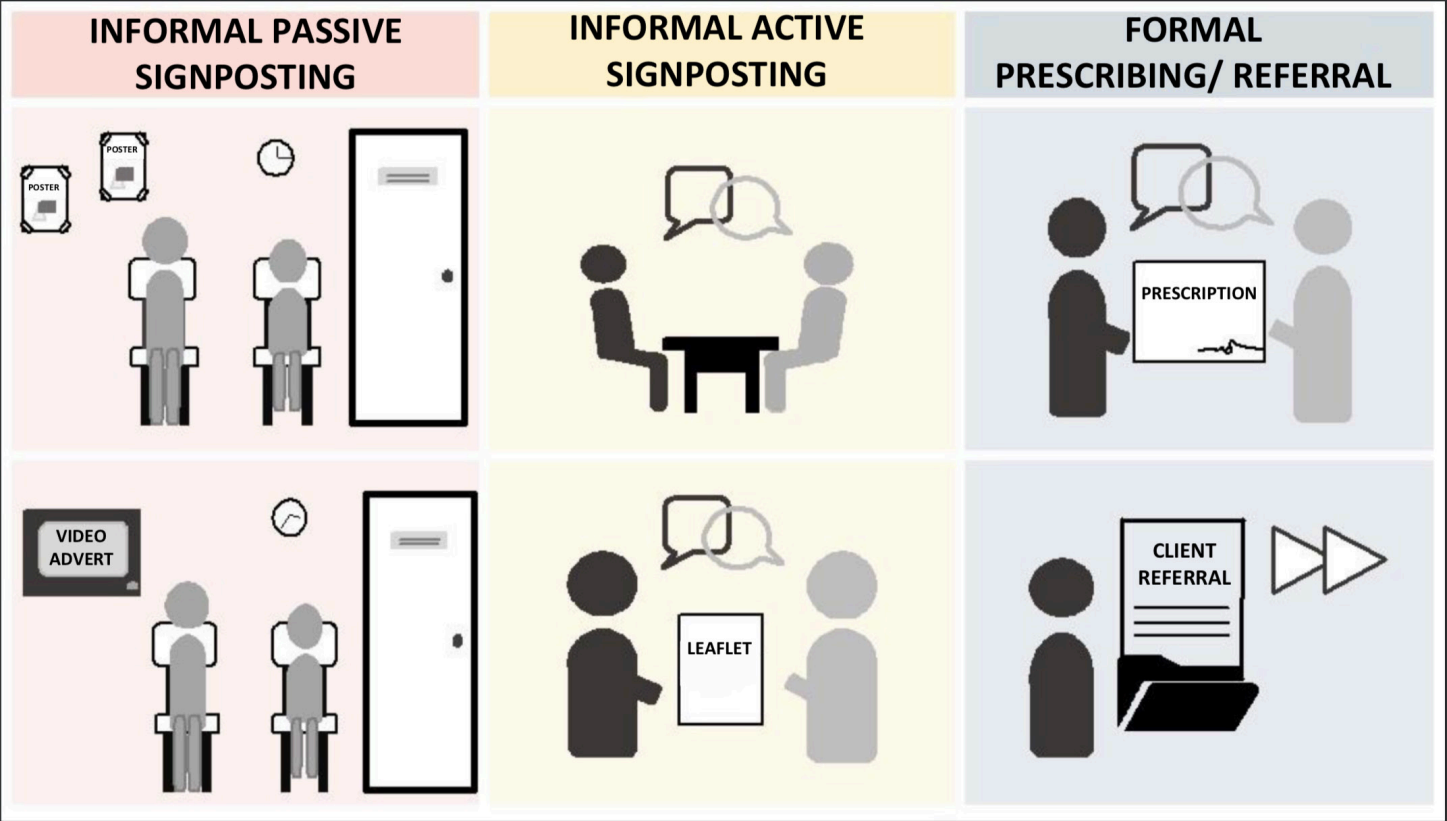
Three potential methods of connecting patients to community-based groups from primary care with examples of how these can be implemented.

Various potential methods of connecting patients to community-based jog**scotland** groups were identified. These could be categorised into three methods; namely, informal passive signposting, informal active signposting, and formal referral/prescribing, based on the type and level of workload associated with the processes of connection. Each of these methods can be implemented in multiple ways (Figure 1). The workload associated with the different methods varies for both HPs and patients; for example, passive signposting has a low workload for HPs as they do not have to actively signpost or refer/prescribe, but a higher workload for patients as they have to decide that the opportunity advertised is relevant for them, and will need to self-refer to seek further information.

Both patients and HPs acknowledged that advertising local PA opportunities, such as jog**scotland,** could easily be achieved at the GP practice, as well as throughout the wider community. The use of posters, leaflets, and television monitors in the practice waiting areas could provide a passive means of sharing knowledge of what is available locally and what the group involves:

*'Like a short video, you know how you can run a rotational thing in practices? If people are just sitting in a waiting room, instead of them just sitting there, they’re watching a 30 second,* *“welcome to jog* ***scotland*** *, this is what we do”* *.* *’* [HP, 52 years, male]

Patients and HPs additionally discussed the formal prescribing of PA:

*'People like to have something in their hand to go out the door with, and so, often, from our point of view, that means a prescription for a drug. If we can give them a prescription for something that isn’t a drug, that would be a good thing.*' [HP, 42 years, male]

For HPs, referring patients on to an intermediary (for example, link worker or exercise co-ordinator) to discuss their PA options personally and in more detail was an attractive method:

*'if we were able to refer somebody* [to] *a physical exercise coordinator, who was then able to go through with a patient the types of problems they have, the types of things they like doing, how they would like to change, what sort of exercise they’d like to do, and then give them a structured bit of advice*.' [HP, 42 years, male]

It is noteworthy that, when discussing being *‘* *referred* *’* by a HP, patients often described examples of informal active signposting, where the HP speaks to them about increasing their PA levels, and prompts them towards different types of activities or resources. Within the method of active signposting, ‘just having the conversation’ and the use of written information as a means of emphasising the conversation was discussed. This was seen to allow people to consider their options and remind them that they can self-refer:

*'I think contact, you know a leaflet and contact card or something like that, would be preferable to just verbally told about it because it jogs, you know jogs the memory when you get home you take it out your pocket and go right, I’ll do something about that*.' [Patient, 34 years, male]

### Barriers and facilitators to the identified methods of connection

Emergent domains and the sub-themes are described below with illustrative quotations in Table 2 (HPs) and Table 3 (patients).

**Table 2.** HP quotations for emergent domain themes and sub-themes

**TDF domain**	**Sub-theme**	**Quotation**
Memory, attention, and decision processes	Patient–HP interaction	*'So, when somebody’s decided their condition requires them to go to a doctor and they’re in front of a doctor then I can certainly raise it. But I don’t usually push it at people until they come to me and say, ‘well, listen, you know’, and then that gives me the ideal opportunity.*' [HP, 50 years, male]
Beliefs about consequences	Patient engagement	*’it’s getting the time, from what I understand, it’s getting the patient at the right time, when they’re motivated, when they're ready to take some change.*' [HP, 45 years, female]*'You are trying with these people, but a lot of them, I think they’re looking for that medication, rather than to engage with others and do self-help.*' [HP, 53 years, female]
	Patient confidence and ability	*'I think for physical activity, like say the [medical condition-specific physical activity programme], and the cardio gym I think sometimes people feel that exercise isn’t for them.'* [HP, 53 years, female]*'There’s the cost thing as well, most people don’t seem to have that much money to go join a gym or to a regular class or sign up to a running club. There’s all that.*' [HP, 49 years, female]*'There are people who struggle to access things that involve travel or effort or being organised.*' [HP, 50 years, male]
Knowledge and environmental context and resources	Time	*'That’s the irony of it, you know, frontline healthcare professionals who are working to 10-minute consultations, you struggle with the accessibility and currency of information*.' [HP, 50 years, male]
	Access and currency of information (awareness of opportunities)	*'I think there’s probably an opportunity with community health and social care hubs, that’s part of what they could potentially do is to signpost people and keep the intelligence on what’s available and what does it do.*' [HP, 50 years, male]*'I guess the other thing is to have champions in each practice. And that wouldn’t necessarily need to be a clinician. It could be people in admin. Or you could have, you know, more than one. So, people who, you know, could disseminate some information and stuff to the others. That would be quite good, wouldn’t it?*' [HP, 56 years, female]
Social/professional role and identity	Position of influence and responsibility	*'I think health professionals have a responsibility to do that. I don’t think we’re the only people that can do it, and I don’t think it should be our sole task or job, but I think there’s an opportunity there, if someone comes along with something that could be helped, or… By improving physical activity, or it could be, in fact, triggered by not being physically active, I think there’s a responsibility to bring that up.*' [HP, 42 years, male]
	Advocate in wider society	*'I think of it as take it out of the medical practice. De-medicalise it, make it part of normal life, okay it was me that triggered it but unshackle the medicalisation of it.*' [HP, 50 years, male]
	Medico-legal responsibility	*'How do I know I’m referring to something appropriate and not a danger to my patients*?' [HP, 38 years, male]

H = health professional. TDF = theoretical domains framework.

**Table 3.** Emergent COM-B components, sub-themes, and quotations from patient analysis

**COM-B component**	**Sub-theme**	**Patient quotation example**
Motivation	HP as facilitator/role of influence	*'Aye, when he sort of brought it up* [discussion on improving physical activity] *I was, sort of, went home and I was thinking to myself, I was like my jeans are a bit tight on me. And I just started noticing things like that. Then I was like* *“* *right I’m going to do something about it* *.* *”* *Give myself something to aim for.*' [Patient, 33 years, male] .*'Think I’d be more encouraged to do something like that, them* [HP] *saying,* *“* *you need to increase your walking* *“* *. I would maybe say,* *“* *okay, I’ll take the dogs out five days a week and that will increase my walking by two and a half times* *”* *,* *or* *“* *I’ll make sure I go for a walk every weekend for two and a half…* *”* *And that’s something you would commit to, because the doctor has said to you, you’ve got to do that.*' [Patient, 64 years, female]
	Legitimacy of action	*'I think it’s something I would be more inclined to try if I was sort of referred to it. I know that sounds ridiculous…I don’t know. It’s hard to put into words. I think it would just, it sounds silly, but I would just feel more justified in going along if I was being told to go basically. Although I know we, as human beings, hate being told to do things as well. Maybe not being absolutely dictated to that I had to go, but if I was referred to it, I’d feel it was just a more legitimate thing to do if that makes sense.*' [Patient, 50 years, female]
	HP manner and approach to topic	*'I think, I think you have to sort of be careful on what you’re doing on that side of things. Because if you have got people that’s on a bit of a downer and that as well, then the fact that you’re sort of putting that across to them as well that* *“you need to lose a bit of weight* *“* *or anything like that, then that could sort of trigger more off. You could get people going a* *way and they could start sulking more. And thinking* *“* *that doctor’s called me fat* *”* *.* *'* [Patient, 33 years, male]*'I suppose, getting people… it’s putting the message across without making people feel guilty for not doing exercise, is one of the most important things*.' [Patient, 43 years, female]
Opportunity	Providing tangible option	*'Having something tangible that the GP’s group can recommend,* *rather than,* *“* *we think you should get a bit more* *physical activity* *”* *.* *'* [Patient, 68 years, female]
	Meet and greet	*'A meet and greet might be good then I wouldn’t mind going along to that on my own. If there was maybe other people going at the same time I’d think,* *“oh, we’re all joining together that’d be fun.* *” And the chances are you might see someone you recognise so that meet and greet might be okay.*' [Patient, 63 years, female]
	Need for social support (buddy system)	*'I was going to say, not that I’ve ever been to Alcoholics Anonymous, I know I’ve got a bit of a food addiction, but I know they’ll have their sponsors. So maybe they could buddy up with somebody who really does take a keen interest in where you’re at, and wants to help you monitor your progress, motivate you, and all the rest of it, then that might be quite an idea.*' [Patient, 50 years, female]

COM-B = capability, opportunity, motivation, behavioural model. HP = health professional.

### HP views

**Figure 2.**
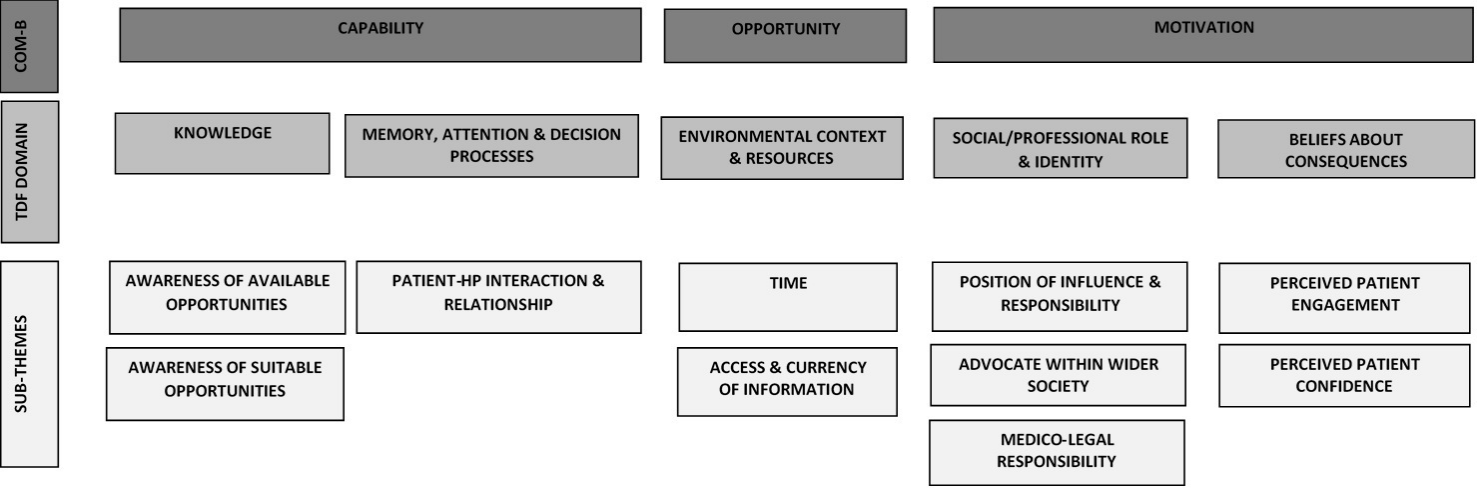
HP barriers and facilitators to connecting patients to community-based physical activity opportunities COM-B = capability, opportunity, motivation, behavioural model. HP = health professional. TFD = theoretical domains framework.

For HPs, the barriers and facilitators for connecting patients to PA opportunities, such as jog**scotland**, fell within five TDF domains mapping across all components of the COM-B model (Figure 2).

#### ​Memory, attention, and decision processes

Real-time decision-making on whether to raise the issue of PA with their patients was guided by how the interaction unfolded and their rapport with the patient during the consultation. This decision involved waiting for an opening when the patient establishes for themselves that PA could help improve health complaints, so would be patient-led. However, HPs acknowledged that this patient-driven approach does not always work. Thus, HPs are the main instigators of discussions concerning PA, and whether or not they decide to do so is often determined by their perception of the patients’ receptivity.

#### ​Beliefs about consequences

Many of the HPs expressed that discussing PA with their patients depended on their beliefs on patients’ engagement and confidence in making improvements. In particular, whether patients would action their suggestion was a significant consideration when deciding on raising the issue during a consultation. HPs also described that many patients did not think that the PA opportunities available were for them, in particular jog**scotland** could be perceived for ‘runners’ only. This perception of the patient’s intentions and beliefs towards PA were also considered alongside the HPs’ perceptions about the barriers for their patients, such as lack of time, availability, accessibility, and suitability.

#### ​Knowledge, environmental context, and resources

HPs identified lack of knowledge and time to discuss PA and different opportunities with patients as a barrier. Access to resources advising what options are available, and time to seek out this information is a critical barrier for HPs. To help overcome these barriers, HPs often described the need for up-to-date resources, and alternative connecting solutions that rely on an intermediary or resource including practice champions, link workers within practices, and community hubs.

#### ​Social/professional role and identity

HPs acknowledge their perceived position of influence and responsibility can be utilised to positively motivate patients towards making improvements. However, HPs often raised the point that clinicians should not be solely responsible, and in fact responsibility lies within the wider community and society to normalise not medicalise.

For some HPs, there was a medico-legal concern for connecting patients to local opportunities such as jog**scotland**, where the HPs lacked knowledge about the suitability and content of these local groups.

### Patient views

For patients the barriers and facilitators identified fell within the COM-B components of motivation and opportunity.

#### ​Motivation

The majority of patients described being open to PA discussions with their HP. Patients acknowledged that a discussion can trigger the little push towards them thinking and actioning on the suggestion to improve their activity levels. Patients share the view with HPs that the HP is in a position of influence, and can act as a motivator and facilitator by connecting them to options. Importantly, patients often discussed the dislike of being dictated to and that, in particular, formal prescribing of PA may not be always be taken positively by some patients. In contradiction however, many patients discussed the legitimacy of being ‘referred’ to something by their HP. Central to this belief is the importance patients place on the ability of HPs to link the benefits of improved PA to their health and/or medical conditions combined with the way they approach the topic.

#### ​Opportunity

Patients often liked the option of being connected to resources on specific PA opportunities by their HP for them to consider and potentially follow-up on. Patients described that connecting to tangible options is favourable because they perceive it as helping them towards implementing the changes instead of just being told ‘you should get more active’.

Some participants suggested a ‘meet and greet’ with organisers and members of a jog**scotland** group in their area. This ‘meet and greet’ (held at a local community location or health centre) could provide the opportunity to ask questions about what is involved and to meet with people before turning up for the first time, a barrier often mentioned by many when they consider starting or turning up to a PA opportunity. Having the social support to go along to one of these jog**scotland** groups was often mentioned by the patients and by the HPs, as acting as a means to help motivate and support patients towards taking the first step towards activity – patients mentioned that a ‘buddy system’ could be useful to help in this support.

## Discussion

### Summary

This study focused on identifying potential methods of connecting primary care patients to local community-based PA opportunities, such as jog**scotland**, and the barriers and facilitators to employing those methods of connection. The study identified three types of methods of connecting primary care patients to local jog**scotland** groups*:* informal passive signposting, informal active signposting, and formal referral or prescribing. The findings confirmed many of the barriers for HPs from previous literature,^18,19,40–43^ which fell within domains of the TDF: knowledge; memory, attention, and decision processes; environmental context and resources; social/professional role and identity; and beliefs about consequences. This study further builds on this knowledge by providing an understanding in the context of community PA opportunities and of patient views on the barriers and facilitators, as well as potential solutions suggested by the HPs and patients for overcoming perceived barriers to connecting patients.

### Comparison with existing literature

Participants discussed various potential methods of connecting patients to community-based PA opportunities, with assorted ways of implementing the methods suggested. What is apparent from the discussions is that no single method was deemed ‘best’. Both ‘actors’ highlight the necessity for a variety of means to make connections to accommodate individual preferences. These methods of connection can differ in their level of HP workload, from passive signposting approach at the practice level, to more formal prescribing or referral. Importantly, within and across these methods, the level of workload for the patient can also vary from a low level of referral and follow-up by others to a higher-level workload of self-referral and active follow-up.

The diversity of methods and workloads for both ‘actors’ in PA connection reflects individualistic needs and wants, as well as beliefs related to whose responsibility it is to ‘do something’ about improving PA. HPs and patients highlighted that linking PA promotion with clinical consultation is key for potential and opportunistic intervention. Nevertheless, and mirroring previous findings,^19,44^ many HPs and some patients acknowledged that the responsibility should not be limited to the HP’s role, rather lying with individuals and with wider societal norms about self-management. This body of evidence reiterates that there is a shared responsibility in health promotion, with HPs and patients alike indicating a desire for and acceptance of connections from HPs to non-medical support for self-management (social prescribing).

The findings point to the ‘motivation’ aspect of the COM-B model of behaviour in terms of what impacts on patients taking up offers of being signposted to community PA opportunities. Mirroring previous findings,^19,45^ both HPs and patients see the role of the HP as a facilitator, but not dictator, with the perception of the HP as a key person of influence with professional responsibility. Patients often contradicted themselves reporting that they did not want to be ‘parented*’* but also reflected that being encouraged or directed by their HP to make changes to their PA was an influential and motivational factor in making changes. In effect, patients described a need to strike a balance between directing and suggesting in a supportive manner, and being too prescriptive — an important aspect for implementation of behaviour change techniques^46^. The acknowledgement and action of the HP raising the topic of PA improvement provided patients a legitimacy to the issue and an opportunity to do something about the problem. The authors identified and categorised methods of connecting to reflect the implementation workload for the HP . However, from a patient’s perspective, the very nature of a HP connecting them to PA opportunities across any means of signposting or referral, was seen as ‘formal’ acknowledgement of the problem. The three modalities are not mutually exclusive, and all three may be beneficial for some people.

The HP–patient relationship and the manner in which HPs raise the topic of PA was also an important consideration raised. Timing was of key importance, ‘*getting the patient at the right time’* (HP, 45 years, female) and getting them motivated^19,43^ to the suggestion of PA improvement was a focal part of the HPs decision-processing. How the topic was raised and linked to patient’s specific health conditions was central to patient acceptance to the topic, supporting previous views on health promotion in healthcare.^45^ Furthermore, providing patients with tangible opportunities to look in to, in contrast to ‘you should get more exercise*’*, was a preferable and more effective approach. However, HPs accessing or having the knowledge of different local opportunities was a major barrier to being able to achieve this.^47^ Being able to provide up-to-date information on an assortment of opportunities was sought. With concern about the medico-legal aspects of connecting patients to groups with which they were not familiar, HPs identified that a solution to their lack of time and knowledge would be for an intermediary (for example, practice champion, community hub, or link worker) to be available locally, to whom they could signpost or formally refer patients, providing — what other literature has described as a bridge between primary care and third party groups.^47^ This type of resource and example of SP was also seen to be a key solution to alleviate time pressures within a consultation, provide practical resource support, and supports the consensus that patients can self-refer and take responsibility for their own health improvement.^48^

Similar to findings by Flannery *et al* *,* ^49^ social support was acknowledged in terms of creating ‘opportunity’, which is reflected in this study's findings with suggestions from patients such as a ‘buddy’ system. HPs and patients discussed that an opportunity to meet *‘*people like me*’*, who are also trying to engage with an opportunity, would be a supportive solution that the practice and wider community could implement. In particular, providing an opportunity to ‘meet and greet’ members and organisers of local jog**scotland**, and other groups, can provide the chance to build relationships and provide the opportunity for patients and HPs to be linked to a tangible option where they can ask questions about what is involved. In particular, this can provide reassurance for HPs on the set-up, qualifications of the leaders, and whether these groups are suitable for their patients.

### Strengths and limitations

This study provided the unique opportunity to explore both actors in health promotion and in gaining an understanding of how different methods of connection impact on the workload associated with the connection for both the HP in their implementation and for the patient in their actioning. Utilising the COM-B model provided a useful framework in understanding key determinants of health-related behaviour and how primary care professionals can play an important role in providing opportunity and motivation for patients in PA improvement. Utilising the TDF for HPs’ views provided a valuable means to understand the individual and organisational determinants of the HPs behaviour and decision-making. Previous research using COM-B and TDF in the PA context appear within the domains of preconception PA guidance and promotion by GPs and community pharmacists,^50^ PA among postnatal women,^51^ and determinants of PA with overweight/obese pregnant women.^49^ This study targeted a more general primary care population with few restrictions in terms of recruitment, and also had the advantage of investigating the views of patients and HPs. As these aspects are vital for the implementation of evidence-based practice,^38,39^ the identification of the relevant TDF domains was fundamental in understanding the potential solutions to making connections in context. Both the interviews and analysis were conducted by members of the research team who have no role within primary care and no relationship to either the HPs or patients who took part in the study. It is believed that participants were forthcoming in their views, with no hesitancy in revealing any negative opinions on the topic.

It is crucial to highlight that the HPs interviewed for this study self-reported frequent PA levels, and thus may be more likely to signpost or refer their patients to PA opportunities.^18^ Furthermore, the HPs in this study may not be a truly representative sample of HPs throughout the NHS due to their keen interest in the study and topic of PA promotion. Equally, there may have been a response bias with patients who were interested in the topic of PA and promotion, thus caution should be implemented in generalisation of the findings.

### Implications for research and practice

PA promotion using connection to community-based opportunities, such as jog**scotland**, was seen by both primary care HPs and patients to be of value. The variety within and across the identified methods of connection highlight the diverse and individualist needs and wants of HPs and patients for PA promotion opportunities.

These findings suggest that health systems that wish to support HPs to deliver PA promotion are likely to benefit from a focus on:

- resource solutions, for example, access to an intermediary person or community information hub to provide information on a variety of different and tangible opportunities
- practice-linked social support for patients through meet and greet, or buddy systems.

Links between jog**scotland** groups and their local GP practices could enable HPs to connect their patients to a tangible PA option. For example, jog leaders and group members hosting ‘meet and greet’ sessions at the practice could allow HPs to gain knowledge of the structure and suitability of this option. This would provide an opportunity to signpost patients to group members from whom patients could seek more information, and get support and reassurance from those who have previously taken up the PA opportunity, as well as establish a ‘buddy’ to start the activity journey with.

Future research should advance the current work and findings by defining and implementing the identified methods of connection from primary care to community-based PA. These intervention models should then be evaluated for acceptability and effectiveness from a wide range of the perspectives, including patients, HPs, community-based groups, and potential implications on the healthcare system. This can provide an understanding for translating the findings for other community-based opportunities.

## Data statement

The datasets used and/or analysed during the current study are available from the corresponding author on reasonable request.
